# Neutrophil activation and neutrophil derived neutrophil extracellular trap formation in patients with coronary artery ectasia

**DOI:** 10.1186/s12872-020-01398-0

**Published:** 2020-03-02

**Authors:** Yuchao Guo, Ruifeng Liu, Lianfeng Chen, Wei Wu, Shuyang Zhang

**Affiliations:** 1 Department of Cardiology, Peking Union Medical College & Chinese Academy of Medical Science, Peking Union Medical College Hospital, No. 1 Shuai Fu Yuan, Beijing, 100730 China; 2 Department of Cardiology, Beijing Friendship Hospital, Capital Medical University, No. 95 Yong An Road, Beijing, 100050 China

**Keywords:** Neutrophil, Neutrophil extracellular traps, Coronary artery ectasia

## Abstract

**Background:**

This study investigated neutrophil activation and neutrophil-derived extracellular traps formation in coronary artery ectasia.

**Methods:**

We enrolled 90 patients who underwent coronary angiography, and included 30 patients with coronary artery ectasia (CAE), 30 patients with obstructive coronary artery disease (CAD) and 30 patients with normal coronary arteries (CON). Intra-neutrophil mean myeloperoxidase index (MPXI) was determined using an automated blood cell counter (ADVIA2120 Hematology System). Serum concentrations of plasma adhesion molecules, cytokines, and neutrophil-derived extracellular traps were quantified.

**Results:**

The intra-neutrophil mean myeloperoxidase index was reduced in CAE patients compared to CAD and CON patients (1.02 ± 3.01, 3.22 ± 3.03, 3.52 ± 4.25, respectively; CAE vs CAD, *p* = 0.016 and CAE vs CON, *p* = 0.007). Multiple logistic regression analysis showed that MPXI and dsDNA were independent factors that predicted the presence of CAE. CAE patients had higher levels of plasma adhesion molecules (P-selectin glycoprotein ligand-1, E-selectin, L-selectin) and interleukin 1 beta levels. Neutrophil extracellular trap concentrations were significantly higher in the CAE group compared to CAD and CON patients (284.31(258.33–449.91) ng/mL, 225.12(203.34–257.13) ng/mL, and 247.37(231.04–273.01) ng/mL, respectively; CAE vs CAD, *p* = 0.000 and CAE vs CON, *p* = 0.001).

**Conclusions:**

Peripheral neutrophils from CAE patients were activated and neutrophil extracellular traps were elevated in the plasma. IL-1β and soluble adhesion molecules may be the causal factors for neutrophil activation.

## Background

Coronary artery ectasia (CAE) is a rare condition that forms a bulge in the coronary arteries that exceeds 1.5 times or more of the normal artery diameter. The underlying pathophysiology remains to be deciphered [1]. Neutrophils are the most abundant immune cells in the body and act as an essential part of the innate immune response [2, 3]. There is increasing evidence that neutrophils and neutrophil-derived products participate in atherogenesis, acute coronary syndrome and coronary artery ectasia [3–6]. To date, the pathogenesis and etiology of coronary artery ectasia are poorly understood, with only a few studies published on the role of neutrophils in CAE [1]. Previous studies have demonstrated that neutrophil serine proteases, neutrophil gelatinase-associated lipocalin and matrix metalloproteinases may be involved in the destruction of the extracellular matrix and development of coronary ectasia [7, 8]. The majority of these proteins are released from different types of granules in neutrophils after neutrophil activation [2]. During this transition process, neutrophils are stimulated by a complicated series of environmental cues including inflammatory bacterial-derived products, host-produced cytokines and adhesion molecules on endothelial cells [2]. Subsequently, neutrophils release their intracellular components. The remnants after the degranulation process form the neutrophil extracellular traps (NETs). NETs have been associated with atherothrombosis, atherogenesis, endothelial dysfunction and experimental abdominal aortic aneurysm [9–11]. To date, relatively few studies have systematically evaluated neutrophil activation in CAE. The current study investigated neutrophil activation and neutrophil-derived NETs in coronary artery ectasia and deciphered its possible mechanistic role.

## Methods

**Fig. 1:**
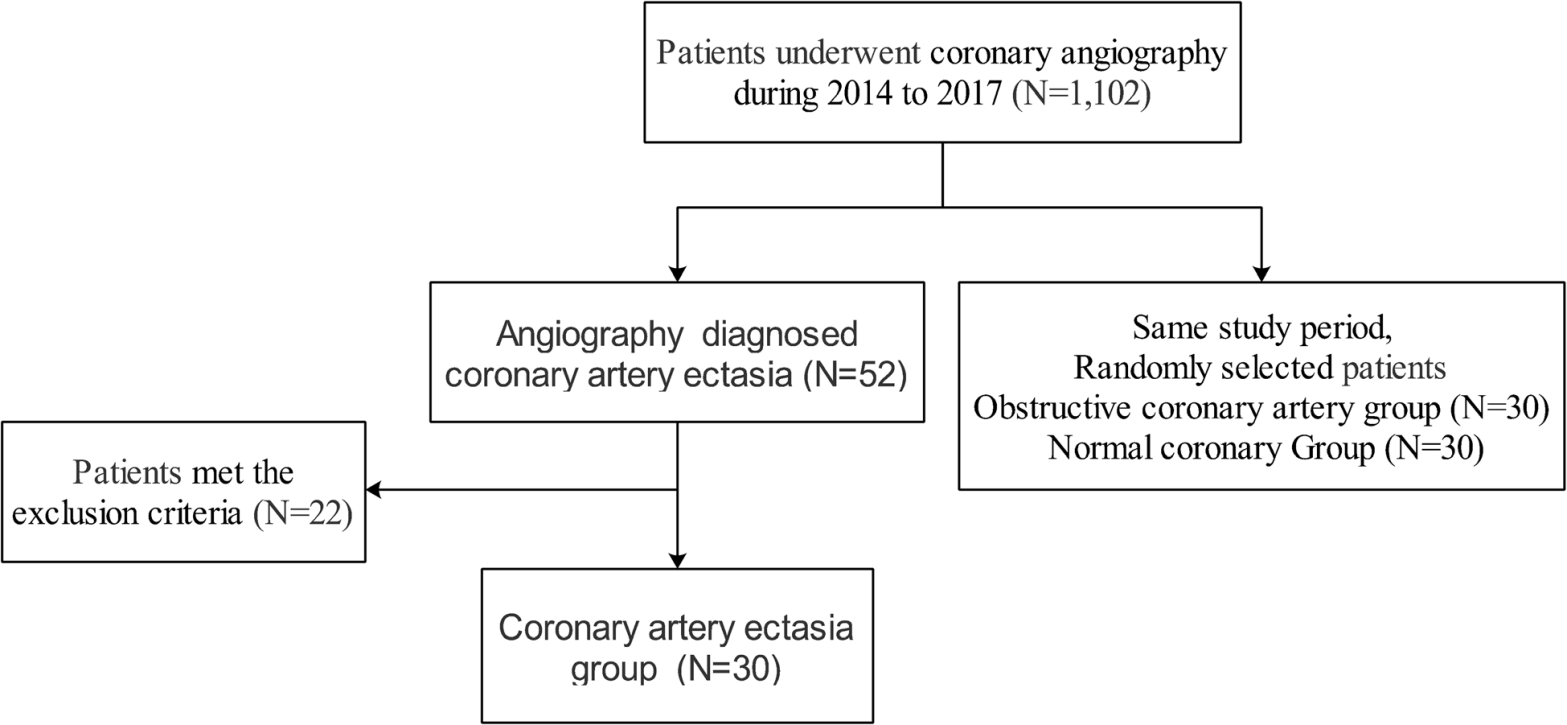
Study flow chart

We prospectively performed a case-control study that included 90 patients without any of the conditions listed in the exclusion criteria between 2015 and 2017 in the catheter room in the Peking Union Medical College Hospital. Blood samples were collected after written informed consent was obtained. Of these patients, 30 patients had coronary artery ectasia and constituted the CAE group. During the study period, 30 patients with obstructive coronary artery and 30 patients with normal coronary angiograms were randomly selected to comprise the CAD and CON group respectively (Fig. 1). This study was approved by the local research ethics committee at Peking Union Medical College Hospital and followed the Declaration of Helsinki guidelines.

The standard Judkins technique was used to perform coronary angiography via the radial or femoral artery and was based on the patient’s peripheral vascular condition (procedure previously described) [12]. The inclusion criteria for the three study groups were as follows. CAE was the abnormal dilation of a coronary artery that exceeded 1.5-fold that of the adjacent normal vessel diameter without apparent stenosis. For patients with diffuse coronary ectasia, an anatomical equivalent coronary artery diameter was considered as the normal value [12]. Four Markis types were proposed in decreasing order of severity based on the location and distribution of CAE: Type 1, diffuse lesions present in at least 2 vessels; Type 2, diffuse lesions in one vessel plus a localized lesion in another vessel; Type 3, diffuse lesions in one vessel; and Type 4, one localized lesion [13]. CAD denoted any type of coronary stenosis> 50% of the diameter of the major epicardial coronary artery with an absence of coronary ectasia. Patients with normal coronary arteries or coronary stenosis < 20% were included in the control group (CON) [13].

Patients were excluded from the study if they had acute coronary syndromes, acute decompensated heart failure, cardiomyopathy, severe valvular heart disease or pulmonary arterial hypertension. Patients with acute infectious diseases, autoimmune diseases (e.g. rheumatoid arthritis, systemic lupus erythematosus), renal failure or cancer were also excluded from this study.

Blood samples were obtained through the peripheral vein using a K2EDTA tube. Blood serum samples were immediately separated by centrifugation and stored at − 80 °C for future experiments. Demographic data and laboratory results were collected from the electronic medical record system. Intra-neutrophil mean myeloperoxidase index (MPXI), which represents the myeloperoxidase content of neutrophils, was determined using an automated blood cell counter (ADVIA2120 Hematology System, Siemens Healthcare Diagnostics) [14].

We quantitatively determined the serum concentration of E-selectin (eBioscience, USA), P-selectin glycoprotein ligand-1 (PSGL-1) (eBioscience), L-selectin (eBioscience), interleukin 1 beta (IL-1β) (BOSTER, Wuhan, China), interleukin 8 (IL-8) (BOSTER), interleukin 17 (IL-17) (BOSTER), and tumor necrosis factor alpha (TNFα) (BOSTER) in 90 patients using commercial ELISA kits. With regards to NET concentrations, PicoGreen (Invitrogen, USA) was used to measure cell-free DNA, which was mainly derived from NETs based on previous publications [15, 16]. Since a gold standard has not been established to measure NETosis, direct measurement of dsDNA was more efficient compared to capture ELISAs for DNA, neutrophil-derived protein complex or image-based flow cytometry [15]. Secretory leukocyte protease inhibitor (SLPI) which inhibits NETs production was measured using a commercial ELISA kit (R&D Systems, USA).

Statistical analyses were performed using IBM SPSS statistics 22.0 (SPSS Inc., USA). Numerical variables with normal distribution were presented as mean ± standard deviation. Non-normal data were presented as median with interquartile range (25th–75th percentile). Categorical data were presented as frequencies and percentages (%). We analyzed the normal distribution of numerical variables using the Kolmogorov-Smirnov test (sample number ≥ 50) or Shapiro-Wilk (sample number < 50) test together with a Q-Q map. We compared continuous variables using t-test and one-way analysis of variance (ANOVA). We compared different variances based on their heterogeneity using the least significant difference (LSD) test or Dunnett T3 test. In addition, we used the Mann-Whitney U test or Kruskal-Wallis H test to determine non-normal distribution variables, and ANOVA with LSD or Dunnett T3 were used in multiple comparison of the rank of non-normal distribution variables. Pearson’s chi-squared test was used to compare frequencies between categories. Univariate logistic regression analysis was used to identify risk markers for CAE. Variables with *p*-value < 0.10 were included for multivariate logistic regression analysis. A final model using multivariate logistic regression was constructed to examine the influence of the following variables on CAE: Diastolic blood pressure, BMI (Body mass index), MPXI and dsDNA. *p* < 0.05 was considered statistically significant.

## Results

**Fig. 2:**
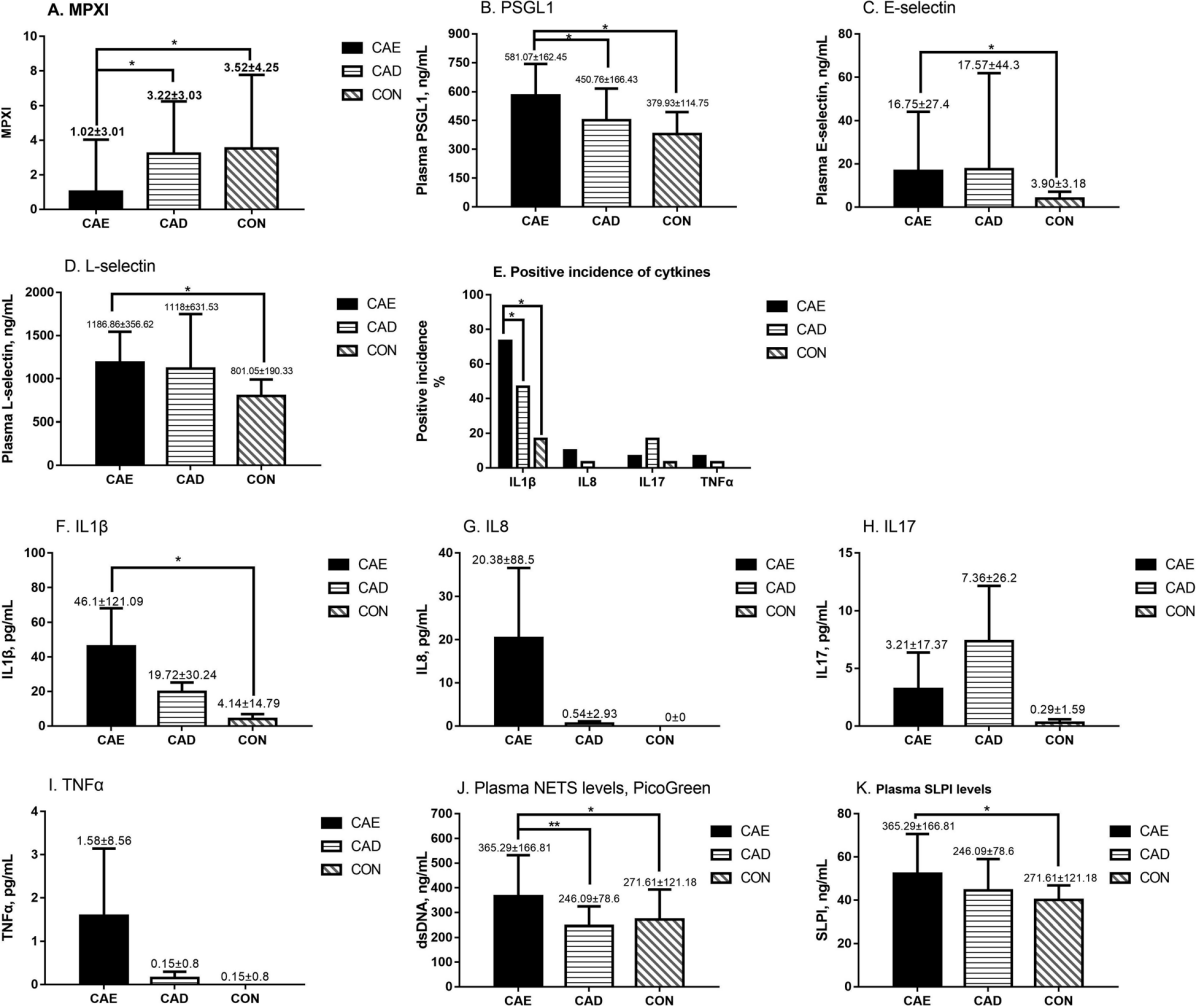
Neutrophil activation, changes in adherence factors and cytokine levels, and neutrophil driven neutrophil extracellular traps for the 3 groups. **a**-**i**: Neutrophil activation marker, adherence factors and cytokine levels in the 3 groups. **j**: Plasma NET levels in the 3 groups measured using PicoGreen. **k**: Levels of secretory leukocyte peptidase inhibitor (SLPI), an endogenous NET inhibitor, in the 3 groups

**Fig. 3:**
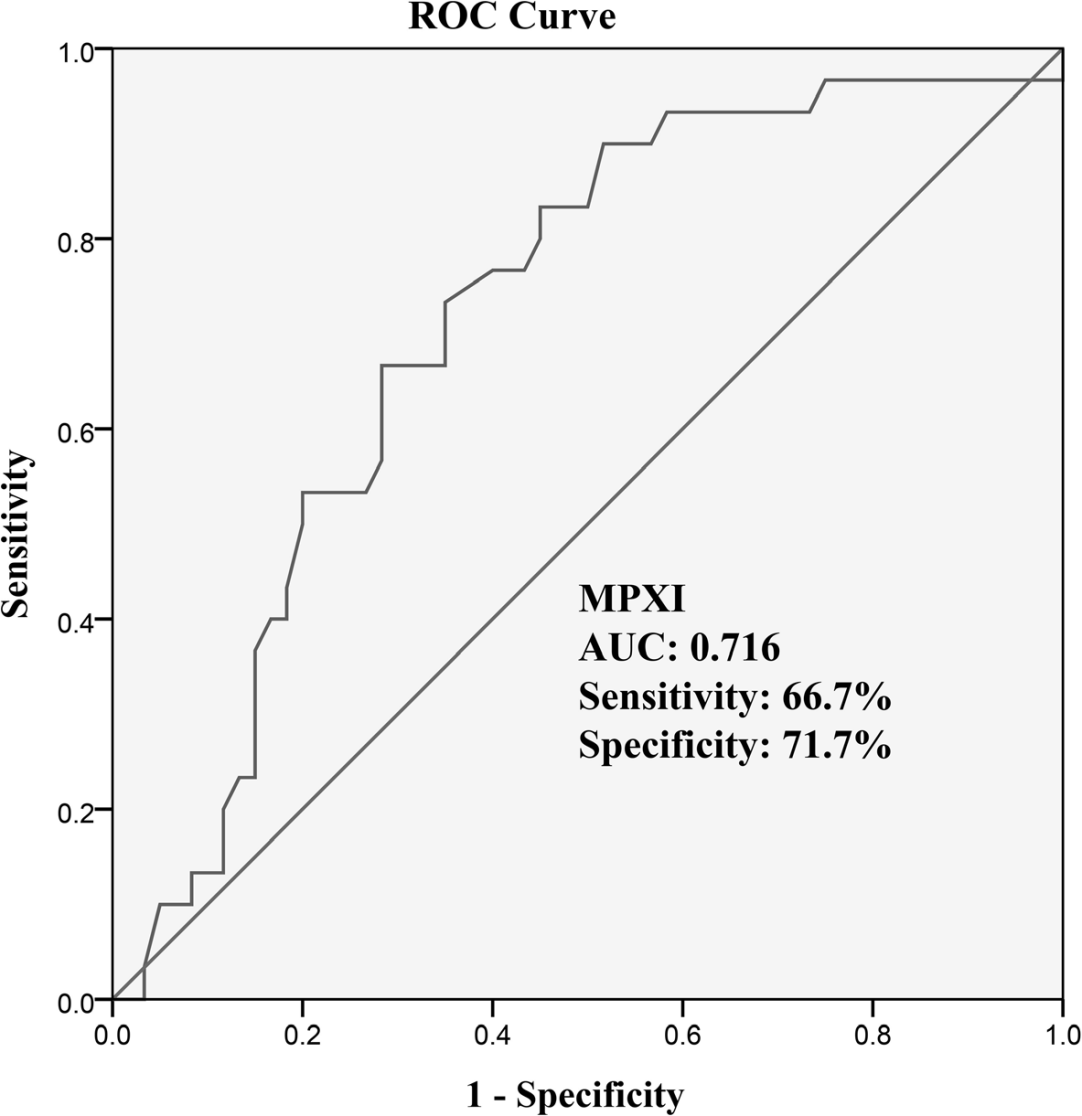
ROC curve analysis of MPXI for predicting isolated CAE (isolated CAE versus non-CAE patients)

The relationship between neutrophil activation markers and angiographic characteristics was investigated. Comparison of demographic data, laboratory results and angiographic characteristics of the study participants are shown in Table 1. We only found differences in hemoglobin between the CAD and CON groups, however, the median for all three groups was within normal ranges. We found no significant differences in Neutrophil-to-lymphocyte ratios in the different groups CAE, not shown in Table 1 (3.45 ± 2.18; CAD: 2.64 ± 1.61; CON: 2.97 ± 4.25, *p* = 0.559). Patients with CAE had a lower MPXI value (1.02 ± 3.01) compared to patients in the CAD or CON groups (3.22 ± 3.03, 3.52 ± 4.25, respectively; CAE vs CAD, *p* = 0.016 and CAE vs CON, *p* = 0.007) (Table 1 and Fig. 2a). Univariate logistic regression analysis showed that diastolic blood pressure, BMI, MPXI and dsDNA were associated with the presence of CAE (Table 2). Multiple regression analyses, which included diastolic blood pressure, BMI, MPXI, and dsDNA showed that MPXI and dsDNA were independent factors for CAE. No significant differences were observed for MPXI between the different Markis types in the CAE cohort (Markis types I: 1.05 ± 4.37; Types II: 0.86 ± 2.13; Types III: 1.08 ± 2.39; Types IV: 0.98 ± 2.28; *p* = 0.999). The area under the curve (AUC) of MPXI in receiver operating characteristic (ROC) analysis was 0.716 (95%CI: 0.612–0.806, *p* = 0.0001, cut-off = 1.7) (Fig. 3).

**Table 1:** Demographic characteristics and laboratory results of the study cohorts

	CAE	CAD	CON	*p* value	CAE vs CAD	CAE vs CON	CAD vs CON
Number of patients	30	30	30				
Age (year)	57.37 ± 13.82	60.8 ± 10.84	56.03 ± 10.15	0.272			
Sex (Female, %)	20.0	33.3	16.7	0.271			
Hypertension (%)	60.0	73.3	43.3	0.061			
DM (%)	16.7	33.3	10	0.067			
Smokers (%)	46.7	46.7	46.7	1.000			
Alcohol consumption (%)	16.7	16.7	26.7	0.535			
Family history of CAD (%)	6.7	6.7	16.7	0.329			
Systolic Blood Pressure (mmHg)	132.27 ± 18.52	129.03 ± 19.12	129.57 ± 17.34	0.766			
Diastolic Blood Pressure (mmHg)	78.73 ± 13.48	74.33 ± 12.12	72.10 ± 11.90	0.119			
Heart rate (bpm)	72.90 ± 12.10	70.63 ± 9.17	69.93 ± 10.47	0.532			
BMI (kg/m^2^)	26.99 ± 3.96	25.36 ± 2.73	25.78 ± 2.73	0.127			
ALT† (U/L)	26(16.75–46.25)	28.5(19.75–37)	22.5(16.75–29.5)	0.336			
Creatinine (μmol/L)	136.69 ± 233.97	79.73 ± 17.15	77.5 ± 12.16	0.166			
LDL-C (mmol/L)	2.34 ± 0.75	2.15 ± 0.70	2.48 ± 0.61	0.185			
WBC# (10^9^cell/L)	6.89 ± 2.25	6.92 ± 1.91	6.59 ± 1.58	0.769			
Neut# (10^9^cell/L)	5.49 ± 2.57	4.36 ± 1.52	5.77 ± 9.28	0.593			
HGB (g/L)	141.07 ± 21.56	135.5 ± 13.95	148.63 ± 15.93	0.017*	0.557	0.333	0.004*
PLT (10^9^cell/L)	211.57 ± 65.15	202.73 ± 63.25	225.57 ± 53.33	0.346			
MPXI	1.02 ± 3.01	3.22 ± 3.03	3.52 ± 4.25	0.013*	0.016*	0.007*	0.745
Markis classification							
Type 1	10(33.3%)						
Type 2	5(16.7%)						
Type 3	11(36.7%)						
Type 4	4(13.3%)						
Ecatatic Coronary artery							
Left anterior descending	19(63.3%)						
Left circumflex	14(46.7%)						
Right	16(53.3%)						

Pearson’s chi-square test was used for frequency differences between categories. ANOVA or Kruskal-Wallis H test (†) was used for continuous variables, LSD or Dunnett T3 was used for multiple comparisons

*CAE* Coronary artery ectasia, *CAD* Coronary artery disease, *CON* Normal coronary artery group, *DM* Diabetes mellitus, *BMI* Body mass index, *ALT* Alanine aminotransferase, *LDL-C* Low-density lipoprotein-cholesterol, *MPO* Myeloperoxidase, *MPXI* Intra-neutrophil mean MPO index, *WBC#* White blood cell, *Neut#* Neutrophil leucocyte count, *HGB* Hemoglobin, *PLT* Platelet count

*Statistically significant

**Table 2:** Univariate and multiple logistic regression analysis displaying independent predictors for isolated coronary artery ectasia

	CAE	non-CAE	Univariate regression		Multiple regression	
			*p* value	OR (95%CI)	*p* value	OR (95%CI)
Number of patients	30	60				
Age (year)	57.37 ± 13.82	58.42 ± 10.68	0.688	0.99(0.96–1.03)		
Sex (Female, %)	20	25	0.598	0.75(0.26–2.18)		
Hypertension (%)	60	58.3	0.880	1.07(0.44–2.62)		
DM (%)	16.7	21.7	0.577	0.72(0.23–2.26)		
Smokers (%)	46.7	46.7	1.000	1(0.42–2.41)		
Alcohol consumption (%)	16.7	21.7	0.577	0.72(0.23–2.26)		
Family history of CAD (%)	6.7	11.7	0.462	0.54(0.11–2.78)		
Systolic Blood Pressure (mmHg)	132.27 ± 18.52	129.30 ± 18.10	0.465	1.01(0.99–1.03)		
Diastolic Blood Pressure (mmHg)	78.73 ± 13.48	73.22 ± 11.96	0.058	1.04(1–1.07)	0.387	1.02(0.98–1.07)
Heart rate (bpm)	72.90 ± 12.10	70.28 ± 9.76	0.271	1.02(0.98–1.07)		
BMI (kg/m^2^)	26.99 ± 3.96	25.57 ± 2.72	0.052	1.15(1.00–1.32)	0.058	1.17(1–1.36)
ALT (U/L)	31.03 ± 18.79	28.33 ± 15.74	0.471	1.01(0.98–1.04)		
Creatinine (μmol/L)	134.9 ± 230.11	78.62 ± 14.78	0.337	1.01(0.99–1.02)		
LDL-C (mmol/L)	2.34 ± 0.71	2.32 ± 0.67	0.888	1.05(0.55–2)		
WBC (10^9^cell/L)	6.89 ± 2.25	6.76 ± 1.75	0.754	1.04(0.83–1.3)		
Neut# (10^9^cell/L)	5.49 ± 2.57	5.07 ± 6.63	0.738	1.01(0.94–1.09)		
HGB (g/L)	141.07 ± 21.56	142.07 ± 16.26	0.804	1(0.97–1.02)		
PLT (10^9^cell/L)	211.57 ± 65.15	214.15 ± 59.14	0.849	1(0.99–1.01)		
MPXI	1.02 ± 3.01	3.37 ± 3.67	0.005*	0.81(0.70–0.94)	0.029*	0.84(0.71–0.98)
dsDNA, ng/mL	284.31(258.33–449.91)	240.16(213.43–270.02)	0.004*	1.01 (1.00–1.01)	0.006*	1.01(1.00–1.01)

*CAE* Coronary artery ectasia, *CAD* Coronary artery disease, *DM* Diabetes mellitus, *BMI* Body mass index, *ALT* Alanine aminotransferase, *LDL-C* low-density lipoprotein-cholesterol, *MPO* Myeloperoxidase, *MPXI* Intra-neutrophil mean MPO index, *WBC#* White blood cell, *Neut#* Neutrophil leucocyte count, *HGB* Hemoglobin, *PLT* Platelet count

*Statistically significant

We then evaluated several typical neutrophil activation related adherence factors and cytokines to determine the underlying cause of neutrophil activation. We found significantly higher serum levels of PSGL1, E-selectin, and L-selectin in patients with CAE (Table 3 and Fig. 2b-d). Correlation analysis for neutrophil activation related cytokines showed that the CAE group had higher IL-1β levels compared to the CON group, while no differences were observed in IL-8, TNFα, or IL-17 levels for the three groups (Table 3 and Fig. 2e-i).

**Table 3:** MPXI and biochemical measurements of the study cohorts

	CAE	CAD	CON	*p* value	CAE vs CAD	CAE vs CON	CAD vs CON
Number of patients	30	30	30				
MPXI	1.02 ± 3.01	3.22 ± 3.03	3.52 ± 4.25	0.013*	0.016*	0.007*	0.745
PSGL1, ng/mL	581.07 ± 162.45	450.76 ± 166.43	379.93 ± 114.75	0.000*	0.001*	0.000*	0.070
E-selectin, ng/mL†	6.49(2.65–17.82)	4.74(1.91–14.32)	3.06(1.45–4.86)	0.036*	0.401	0.011*	0.082
L-selectin, ng/mL	1186.86 ± 356.62	1118 ± 631.53	801.05 ± 190.33	0.002*	0.539	0.001*	0.006*
IL1β, pg/mL† (%)	46.1 ± 121.09 (73.3)	19.72 ± 30.24 (46.7)	4.14 ± 14.79 (16.7)	0.000 (0.000*)	0.296 (0.035)	0.000* (0.000*)	0.018* (0.012*)
IL8, pg/mL† (%)	20.38 ± 88.5 (10)	0.54 ± 2.93 (3.3)	0 ± 0 (0)	0.161 (0.160)			
TNFα, pg/mL† (%)	1.58 ± 8.56 (6.7)	0.15 ± 0.8 (3.3)	0 ± 0 (0)	0.360 (0.355)			
IL17, pg/mL† (%)	3.21 ± 17.37 (6.7)	7.36 ± 26.2 (16.7)	0.29 ± 1.59 (3.3)	0.169 (0.168)			
dsDNA, ng/mL†	284.31(258.33–449.91)	225.12(203.34–257.13)	247.37(231.04–273.01)	0.000*	0.000*	0.001*	0.041*
SLPI, ng/mL	52.24 ± 18.41	44.47 ± 14.57	40.02 ± 6.80	0.004*	0.207	0.005*	0.352

Pearson’s chi-square test was used for frequency differences between categories. ANOVA or Kruskal-Wallis H test (†) was used for continuous variables, LSD or Dunnett T3 was used for multiple comparisons

*CAE* Coronary artery ectasia, *CAD* Coronary artery disease, *CON* Normal coronary artery group, *MPXI* Intra-neutrophil mean MPO index, *PSGL1* P-selectin glycoprotein ligand 1, *IL1β* Interleukin 1 beta, *IL8* Interleukin 8, *TNFα* Tumor Necrosis Factor-Alpha, *IL17* Interleukin 17, *SLPI* Secretory Leukocyte Peptidase Inhibitor

*Statistically significant. Bonferroni correction was used to counteract multiple comparisons between certain categories. Each individual hypothesis was tested at α = 0.05/3 = 0.0167

We also demonstrated that neutrophil-driven NETs were significantly elevated in CAE. The release of cell-free dsDNA into circulation, which is a marker for NETs generation, was significantly higher in CAE patients (Table 3 and Fig. 2j). In addition, the CAE group had higher serum levels of SLPI, an endogenous NET inhibitor, compared to the CON group (Table 3 and Fig. 2k).

## Discussion

Several previous studies have highlighted the importance of neutrophil counts or neutrophil-derived proteases in CAE [17]. However, only a few studies have been published with regards to neutrophil activation and its etiology or consequence in CAE [17]. Our results demonstrated that circulating neutrophils in CAE patients have lower levels of myeloperoxidase, which signifies systemic neutrophil activation during CAE. In addition, higher serum levels of soluble adhesion molecules and IL-1β suggest chronic inflammation in CAE patients, which may lead to neutrophil activation. Our study determined that markers of NETs formation were elevated in coronary artery ectasia and NETs may participate in the progression of coronary artery ectasia.

Recent studies have focused on the contribution of neutrophils, neutrophil-to-lymphocyte ratios, and neutrophil-derived granule proteins in atherosclerosis, coronary artery disease and coronary artery ectasia [3, 17, 18]. In the CAE group, the quality rather than the number of neutrophils was different. Leukocyte activation is essential for neutrophil granule secretion [2]. Neutrophil derived granule proteins, including neutrophil gelatinase-associated lipocalin, matrix metalloproteinases, and elastase, have been reported to be increased in CAE and may contribute to artery media destruction and extracellular matrix turnover [7, 19]. The results from these previous publications are consistent with our findings that neutrophil activation is more predominant in CAE compared to neutrophils counts or neutrophil-to-lymphocyte ratios. As a result, neutrophils in CAE patients are more prone to stimuli, which may exacerbate media destruction. No significant differences in neutrophil activation markers (Fig. 2a) between patients with obstructive coronary artery disease and individuals with normal coronary arteries were observed. These results are consistent with previous studies that demonstrated neutrophil activation may be a unique characteristic for coronary artery ectasia [20]. These results and our ROC analysis suggest that a lower MPXI (< 1.7) is an independent predictor for CAE.

Prior studies have highlighted the importance of inflammation in the development of CAE [1]. Increased levels of all three selectins investigated in this study were consistent with previous findings. E-selectin, PSGL1, and L-selectin promote inflammation by facilitating immune cell activation, while the latter two selectins are crucial for neutrophil and endothelium recognition [2]. The IL-1β pathway plays a central role in the NLR family pyrin domain containing 3 inflammasome, and was designated as an effective therapeutic target for atheroprotection in the CANTOS trial [21, 22]. In addition, CAE patients have been demonstrated to have a larger amount of epicardial adipose tissue, which may produce higher amounts of cytokines, including tumor necrosis factor-alfa and interleukin-1beta [23]. These results are consistent with our findings that the IL-1β pathway is associated with CAE. A significant reduction in cardiovascular events has also been demonstrated after administration of canakinumab, an inhibition of IL-1β. With the evidence of inflammation observed in CAE patients, anti-inflammatory therapy, especially IL-1β inhibition, may be an attractive therapeutic target for CAE.

This study provides the first comprehensive assessment of NET formation in CAE. Several recent studies suggest a role of NETs in atherosclerosis and arterial injury [24]. Effector mediators in NETs, including multiple proteinases and the pro-oxidant enzyme- myeloperoxidase, have been demonstrated to be causal factors for media destruction and endothelial dysfunction, and hence may also exacerbate coronary artery ectasia. Activated endothelium and IL-1β promote or enhance the induction of NETs. Consistent with this, we observed elevated levels of selectins and IL-1β [25, 26]. In contrast, NETs induced by cholesterol crystals can prime macrophages for IL-1β release [5, 26]. There may be an underlying positive feedback process between NETs and IL-1β. SLPI has been shown to inhibit neutrophil elastase and cathepsin G [4]. The central role played by neutrophil elastase is to cleave histones during NET formation. Previous studies have demonstrated that inhibition of neutrophil elastase was an effective method to inhibit NETosis. A possible explanation for the increased levels of SLPI in CAE patients may be that SLPI is stored in azurophilic granules in neutrophils. As a result, neutrophils will inevitably and simultaneously release SLPI together with neutrophil-derived proteins. In summary, activated endothelium and IL-1β may activate neutrophils and increase the production of NETs, thereby inducing CAE progression [25, 26].

This study had several limitations. First, the study cohorts were relatively small due to the rarity of coronary ectasia, however, a post-hoc analysis using G*Power (Universität Düsseldorf, Germany) showed that the ANOVA test of MPXI achieved a power of 0.88. Second, we did not perform follow-up on our study cohort. Additional studies are required to determine the prognostic value of neutrophil activation markers and neutrophil extracellular traps for CAE patients. Third, we only used peripheral blood rather than coronary blood samples. Neutrophil activation is theoretically more predominant in samples obtained around the ectasia area. However, direct sampling from coronary arteries is an invasive procedure and may cause complications. Fourth, this study lacked preclinical data to support the selectins-IL-1β-neutrophil activation-NETs axis. Finally, there may be more accurate markers for neutrophil activation and NET formation, i.e., neutrophil CD11b and CD66b are direct markers for neutrophil activation, however, measurement of these markers requires rapid isolation of neutrophils [20].

## Conclusions

In conclusion, our study demonstrated activation of peripheral neutrophils and NETs formation in CAE patients. The underlying causative factors for neutrophil activation may include IL-1β and soluble adhesion molecules.

## Data Availability

All data analyzed during this study are included in this article. Raw data of the results are available from the corresponding author.

## Abbreviations

BMI: Body mass index
CAD: Obstructive coronary artery disease
CAE: Coronary artery ectasia
CON: Normal coronary arteries, control group
IL: Interleukin
MPXI: Intra-neutrophil mean myeloperoxidase index
NETs: Neutrophil extracellular traps
PSGL-1: P-selectin glycoprotein ligand-1
SLPI: Secretory leukocyte protease inhibitor
TNFα: Tumor necrosis factor alpha
